# Avian Influenza A H7N9 Virus Induces Severe Pneumonia in Mice without Prior Adaptation and Responds to a Combination of Zanamivir and COX-2 Inhibitor

**DOI:** 10.1371/journal.pone.0107966

**Published:** 2014-09-18

**Authors:** Can Li, Chuangen Li, Anna J. X. Zhang, Kelvin K. W. To, Andrew C. Y. Lee, Houshun Zhu, Hazel W. L. Wu, Jasper F. W. Chan, Honglin Chen, Ivan F. N. Hung, Lanjuan Li, Kwok-Yung Yuen

**Affiliations:** 1 Department of Microbiology, The University of Hong Kong, Hong Kong, China; 2 State Key Laboratory of Emerging Infectious Diseases, The University of Hong Kong, Hong Kong, China; 3 Research Centre of Infection and Immunology, The University of Hong Kong, Hong Kong, China; 4 Department of Medicine, The University of Hong Kong, Hong Kong, China; 5 State Key Laboratory for Diagnosis and Treatment of Infectious Diseases, the First Affiliated Hospital, College of Medicine, Zhejiang University, Hangzhou, China; 6 Collaborative Innovation Center for Diagnosis and Treatment of Infectious Diseases, Hangzhou, China; 7 Zhejiang University, Hangzhou, China; Boston University School of Medicine, United States of America

## Abstract

**Background:**

Human infection caused by the avian influenza A H7N9 virus has a case-fatality rate of over 30%. Systematic study of the pathogenesis of avian H7N9 isolate and effective therapeutic strategies are needed.

**Methods:**

BALB/c mice were inoculated intranasally with an H7N9 virus isolated from a chicken in a wet market epidemiologically linked to a fatal human case, (A/chicken/Zhejiang/DTID-ZJU01/2013 [CK1]), and with an H7N9 virus isolated from a human (A/Anhui/01/2013 [AH1]). The pulmonary viral loads, cytokine/chemokine profiles and histopathological changes of the infected mice were compared. The therapeutic efficacy of a non-steroidal anti-inflammatory drug (NSAID), celecoxib, was assessed.

**Results:**

Without prior adaptation, intranasal inoculation of 10^6^ plaque forming units (PFUs) of CK1 caused a mortality rate of 82% (14/17) in mice. Viral nucleoprotein and RNA expression were limited to the respiratory system and no viral RNA could be detected from brain, liver and kidney tissues. CK1 caused heavy alveolar inflammatory exudation and pulmonary hemorrhage, associated with high pulmonary levels of proinflammatory cytokines. In the mouse lung cell line LA-4, CK1 also induced high levels of interleukin-6 (IL-6) and cyclooxygenase-2 (COX-2) mRNA. Administration of the antiviral zanamivir did not significantly improve survival in mice infected with CK1, but co-administration of the non-steroidal anti-inflammatory drug (NSAID) celecoxib in combination with zanamivir improved survival and lung pathology.

**Conclusions:**

Our findings suggested that H7N9 viruses isolated from chicken without preceding trans-species adaptation can cause lethal mammalian pulmonary infection. The severe proinflammatory responses might be a factor contributing to the mortality. Treatment with combination of antiviral and NSAID could ameliorate pulmonary inflammation and may improve survival.

## Background

Avian influenza A H7N9 virus infecting human first emerged in China in February 2013 [1]–[3]. It is associated with a crude case-fatality rate of over 30% in humans despite of oseltamivir treatment [4]. Severe human infections were characterized by rapidly progressive acute community-acquired pneumonia, multi-organ dysfunction and cytokine dysregulation, which did not respond to treatment with antibiotics against typical and atypical pneumonic pathogens [5]. Similar to the influenza A H5N1 and other avian influenza viruses, most patients with H7N9 infection had a history of direct or occupational contact with poultry or visits to wet market [1], [6]–[8]. Phylogenetic analysis showed that this H7N9 virus is a novel triple reassortant virus comprising of hemagglutinin (HA) gene from H7N3, neuraminidase (NA) gene from H7N9 and internal genes from H9N2 [9], [10]. Some of the human isolates already harboured genetic mutations of the polymerase PB2 gene which favour mammalian adaptation, and mutations of the hemagglutinin (HA) gene which increase its binding affinity for human type α-2,6 sialic acid linked receptors [3]. Novel reassortants have emerged in Southern China, which contained additional potential virulence markers [11].

Since most patients had poultry contact and the internal gene segments are closely related to H9N2 isolated from domestic poultries, it has been postulated that H7N9 virus from wild birds first entered the domestic poultry population, reassorted with other avian influenza viruses, acquired the characteristics for adaptation to humans and finally infected humans [3], [12], [13]. One major question is whether H7N9 virus isolated from poultry can directly infect humans. Mice and ferrets have been used as surrogates to study the pathogenicity of the 2013 H7N9 viruses in mammals. Several studies have shown that the human H7N9 virus A/Anhui/1/2013 (AH1), which was isolated from a fatal human case [2], could cause death in mice without prior adaptation [14]–[18], while another human H7N9 virus A/Shanghai/2/2013 did not lead to death in mice [19]. On the other hand, H7N9 viruses isolated from poultries or wild birds appeared to be less virulent in mouse models. H7N9 viruses isolated from one chicken and two pigeons of China in 2013 did not cause any signs of disease [18]. H7N9 viruses isolated from a duck of Japan in 2011 and from a shoveler of Egypt in 2007 caused fatal disease in mice, but the 50% mouse lethal doses (MLD_50_) were much higher than that of the human H7N9 isolates [14], [17].

In our previous study, we have isolated an H7N9 virus from a chicken (A/chicken/Zhejiang/DTID-ZJU01/2013 [CK1]) in a wet market epidemiologically linked to a patient with fatal H7N9 infection [1]. Since the patient likely acquired the H7N9 virus from the market, we postulate that CK1 may cause severe disease in mammals without further adaptation. In this study, we assessed the viral tropism and replication, histopathological changes and the host cytokine/chemokine response in CK1-infected mice. The human virus AH1 was used for comparison. Furthermore, we studied the treatment effect of a combination of neuraminidase inhibitor zanamivir and non-steroidal anti-inflammatory drug (NSAID) celecoxib in a BALB/c mouse model because combination treatment of zanamivir and celecoxib improved the survival of mice infected with H5N1 virus whereas zanamivir alone was significantly less effective [20].

## Results

### Chicken H7N9 virus CK1 caused severe lung inflammation and fatal outcome in mice without prior adaptation

CK1 and AH1 were propagated in chicken embryos, and the viral titres in the allantoic fluid were determined in Madin Darby canine kidney (MDCK) cells. The viral titres were 10^8.2^ tissue culture infective doses (TCID_50_) per ml and 10^7.66^ plaque forming units (PFU) per ml for CK1, and 10^8.8^ TCID_50_ per ml and 10^8.4^ PFU per ml for AH1. Next, we determined the MLD_50_ of CK1 in BALB/c mice, and compared to that of AH1. CK1 caused lethal infection with a mortality rate of 83% (5/6) at inoculation dose of 10^6^ PFU which was the highest dose that could be tested in this study, but no mice died when infected with 10^5^, 10^4^ or 10^3^ PFU. The MLD_50_ dose of CK1 could only be assumed to be between 10^5^-10^6^ PFU, while the MLD_50_ dose for AH1 was determined to be 10^4.8^ PFU. This indicated that CK1 is less virulent in mice than AH1. Since 10^6^ PFU of CK1 and 10^5^ PFU of human AH1 caused similar rate of mortality in mice, we used these doses in the subsequent experiments for the study of pathogenesis.

**Figure 1:**
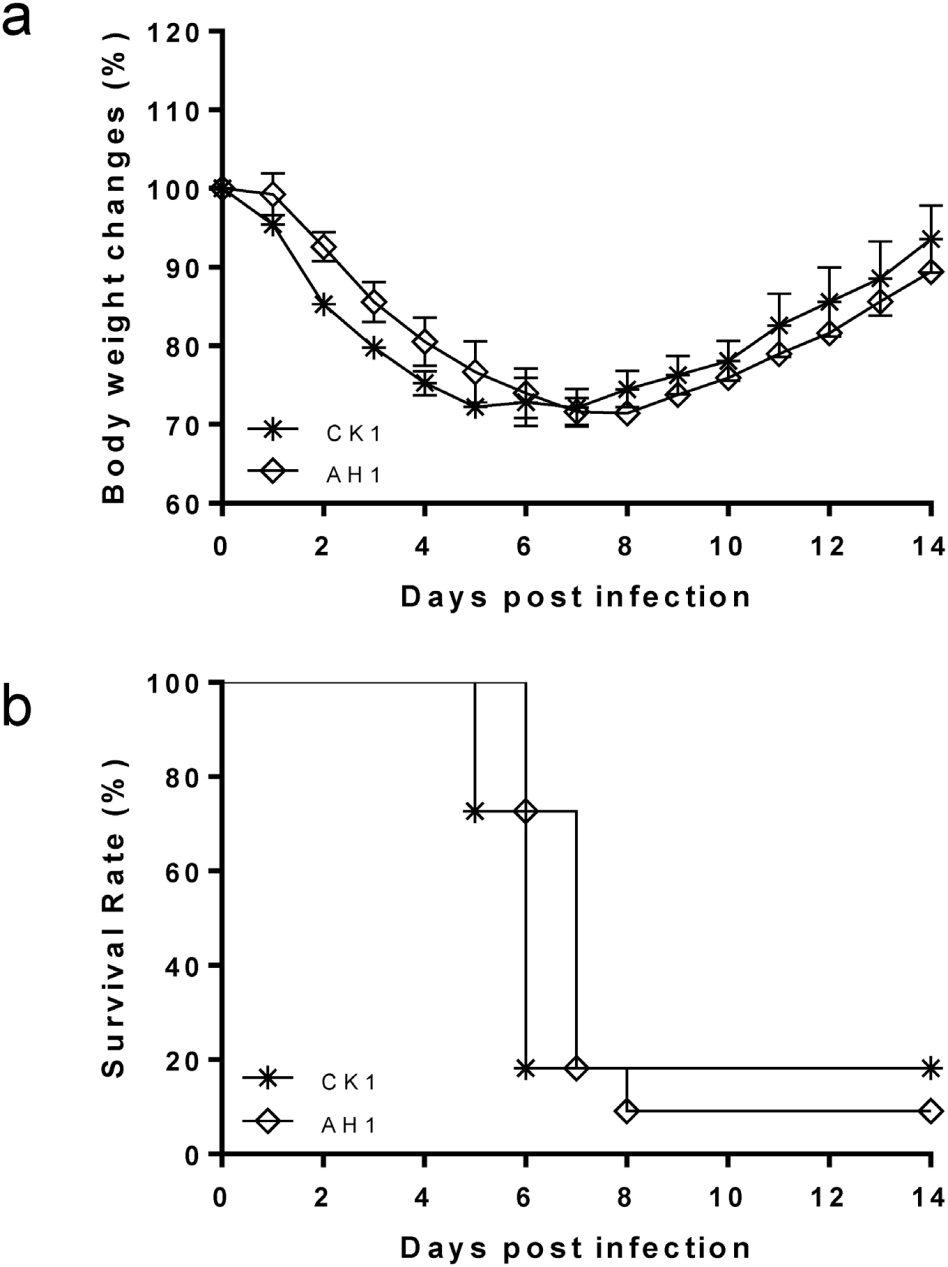
Body weight changes (a) and survival rate (b) of the BALB/c mice infected with 10^6^ PFU of A/chicken/Zhejiang/DTID-ZJU01/2013(H7N9) (CK1, *) or 10^5^ PFU of A/Anhui/1/2013(H7N9) (AH1, ⋄) via intranasal route. Body weight and survival were monitored for 14 days after virus infection. Data shown are the average of three experiments (n = 17 for CK1 and n = 20 for AH1 group).

As shown in Fig. 1, significant morbidity and mortality were observed in CK1-infected mice during the 14-day study period. Two to three days after infection, the mice started to show disease symptoms of ruffled fur before developing laboured breathing and loss of body weight (Fig. 1a). The mortality rates were 82% (14/17) for CK1 and 90% (18/20) for AH1 (Fig. 1b).

**Figure 2:**
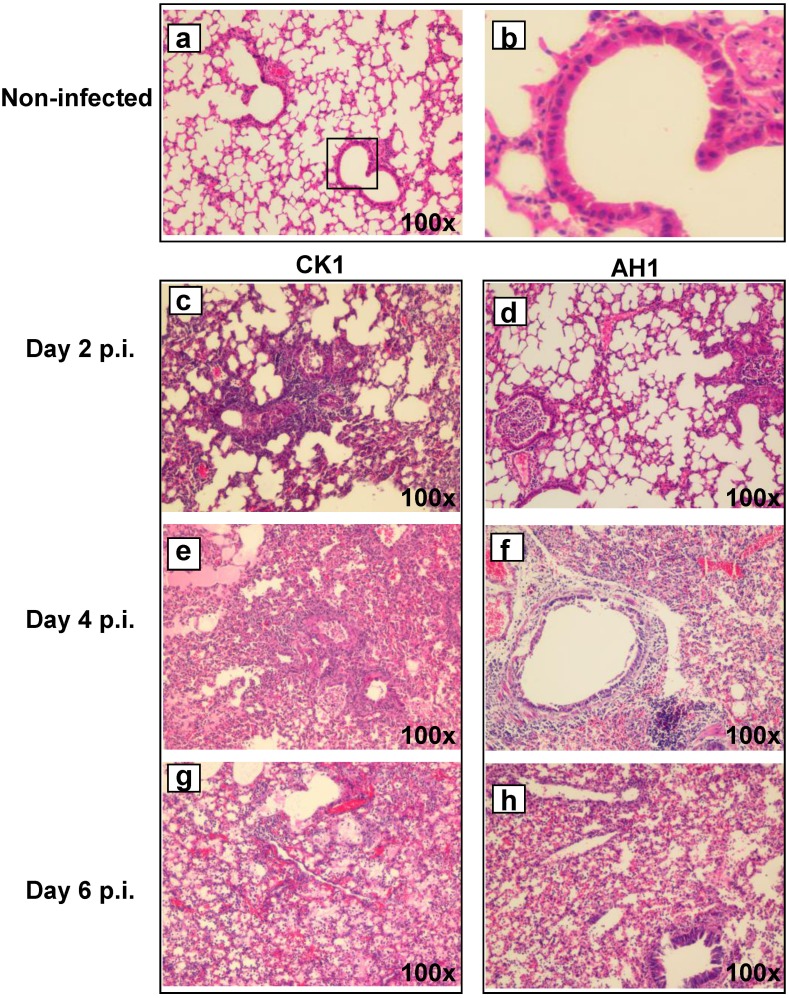
Histopathological changes in the lung tissue infected with CK1 or AH1. Representative histological images of haematoxylin and eosin (H&E) stained lung tissue sections of normal mouse lung (a, and amplified image b) and infected mouse lung at various time points post infection (c-h). Mouse lung at day 2 p.i. showed peribronchiolar interstitial infiltration, bronchiole epithelial cell necrosis and necrotic cell debris within alveolar lumens (c, CK1 infection*;* d, AH1 infection). At day 4 p.i, mouse lung showed alveolar space exudation, bronchiole epithelial cell necrosis, alveolar cell necrosis and destruction of alveolar wall (e and f). At day 6 p.i., alveolar exudation, infiltration, hyaline membrane formation and alveolar hemorrhage with red blood cells within the alveolar space (*g* and *h*). Original magnification ×100.

Histopathological findings of lung tissues from infected mice were examined, scored, and compared with those of non-infected mice (Fig. 2). At day 2 post-infection (p.i.), CK1-infected mouse lungs showed a typical and severe viral pneumonia with focal perivascular and peribronchiolar interstitial lymphocytes, monocyte/macrophages infiltration and vascular congestion. One notable feature was the widely distributed bronchial and bronchiolar epithelial cells necrosis (Fig. 2c and 2d). At day 4 p.i., the inflammatory and necrotic changes affected larger areas of the lung including walls of alveoli (Fig. 2e and 2f). These changes became more severe at day 6 p.i., and were accompanied by alveolar hemorrhage (Fig. 2g and 2h). Pulmonary vascular endothelial damage, vascular thrombosis and perivascular edema could be seen in some of the infected lungs (data not shown). The type of pulmonary pathological changes in CK1 was similar to AH1-infected mouse lungs. There was no significant difference in the semi-quantitative histological scores between CK1 and AH1 infection (Table 1).

**Table 1:** Average histological score of CK1- and AH1-infected mouse lung tissues*.

Histological changes	CK1			AH1		
	Day 2 p.i. (n = 3)	Day 4 p.i. (n = 3)	Day 6 p.i. (n = 3)	Day 2 p.i. (n = 3)	Day 4 p.i. (n = 3)	Day 6 p.i. (n = 3)
**Necrosis**	2.6	3.3	2.6	2.3	3.3	3.6
**Infiltration**	2.6	3.8	4.0	3.0	3.1	4.0
**Hemorrhage**	1.0	2.3	2.6	1.0	1.6	2.0

p.i.: post-infection.

*Details of the histological scores are presented in Table 2.

### CK1 infected multiple cell types in the mouse respiratory tract

**Figure 3:**
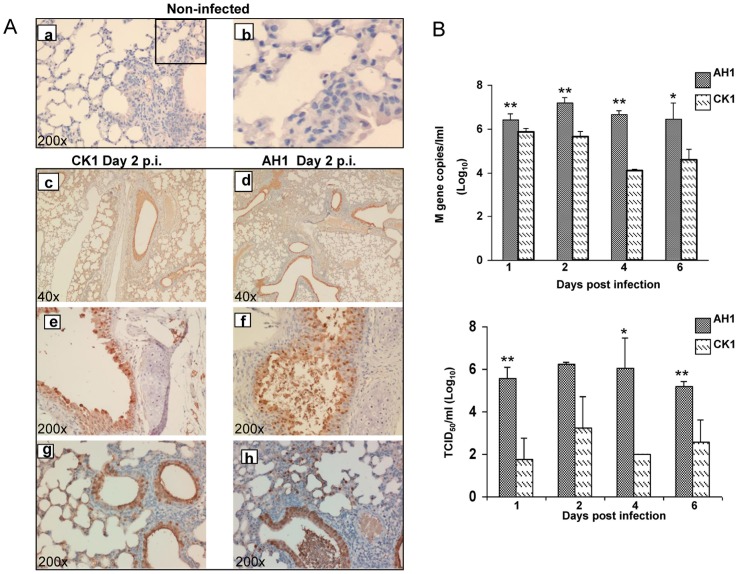
CK1 and AH1 replication profile in mouse lung. A. Representative images of immunohistochemically stained influenza nucleoprotein (NP) in formalin fixed mouse lung tissue infected with CK1 or AH1 at day 2 p.i. Viral NP protein was labeled brown by 3,3′-diaminobenzidine (DAB). Uninfected mouse lung as negative control (*a)*, amplified image (b); Representative images of CK1(c) and AH1 (d) infected mouse lung stained NP positive at 40x magnification. Trachea epithelial cells (e and f), bronchiole epithelial and alveolar epithelial cells (g and h) were stained positive for viral NP protein. (Original magnification ×200). B. Viral load in infected mouse lung homogenates. Mice were infected with 10^5^ of AH1 or 10^6^ PFU of CK1, at day 1, 2, 4 and 6 p.i., 3–5 mice from each group were sacrificed. The left side of the lung was homogenized in 1 ml of MEM culture medium. Viral loads were determined by amplification of viral M gene copy numbers by real time RT-PCR (top panel), and infectious viral titre were determined by TCID_50_ assay on MDCK cells (bottom panel). **P<0.01; * *P*<0.05.

To determine the tissue tropism of CK1, immunostaining was performed using antibody targeting the influenza nucleoprotein (NP). AH1-infected mice were used as the control. Immunostaining of lung tissues from uninfected mice with mouse anti-NP antibody showed that there was no non-specific staining (Fig. 3A, a and b). CK1 and AH1 infected various cell types in the mouse respiratory tract from trachea to the alveoli (Fig. 3A, c to h). NP-positive stainings were seen in the epithelial cells of the trachea (Fig. 3A, e and f), bronchioles and alveolar pneumocytes (Fig. 3A, g and h). Morphologically, NP-positive cells in alveoli were mainly type II pneumocytes. No differences in the distribution and cell types of NP-positive cells were observed between CK1- and AH1-infected lungs. However, even at 10-times higher inoculation dose, CK1-infected mice had a persistently lower pulmonary viral load from day 2 p.i. to day 6 p.i when compared to those infected by AH1 using both quantitative reverse transcriptase-polymerase chain reaction (RT-PCR) and TCID_50_ assay (*P*<0.01 or <0.05, Fig. 3B). This may suggest that CK1 did not replicate as efficiently as AH1 in mouse lungs.

**Figure 4:**
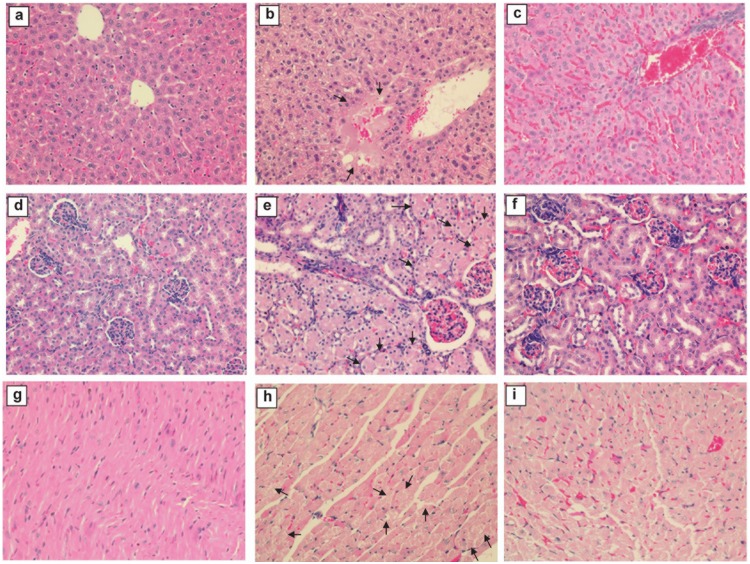
Liver, kidney and heart tissue degenerative changes in CK1-infected mice at day 6 post infection. Representative images of haematoxylin and eosin (H&E) stained tissue sections are presented. The liver (a), kidney (d), heart (g) of uninfected mice were shown for comparison. CK1-infected mice at day 6 p.i. showed liver hepatocytes degeneration, focal cells necrosis (arrows, b) and hemorrhagic changes (c); kidney tubular epithelial cells degenerative changes (arrows, e) and peritubular vessels congestion (f). Mild myocardial cell swelling (arrows, h) and red blood cells infiltrating between degenerative myocardial fibers (i) were seen in the heart. Original magnification ×200.

To determine whether chicken H7N9 virus could disseminate outside the respiratory system, viral RNA detection was performed by quantitative RT-PCR in brain, liver and kidney tissues. No viral genome RNA was detectable in these tissues, suggesting the absence of extrapulmonary viral replication. But on day 6 p.i., degenerative changes in liver, heart and kidney including hepatocytes degeneration and focal cells necrosis (Fig. 4b and 4c), kidney tubular epithelial cells degeneration and peri-tubular vessels congestion (Fig. 4e and 4f), and myocardial cell swelling with red blood cells infiltrating between myocardial fibers, were observed (Fig. 4h and 4i).

### CK1 induced high level of pulmonary proinflammatory cytokines and chemokines

**Figure 5:**
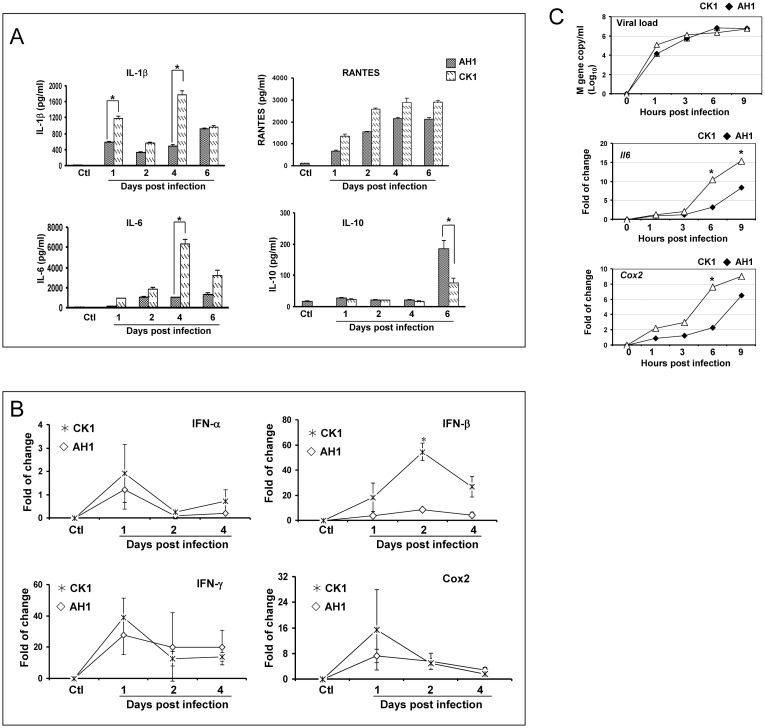
Cytokines and COX-2 expression in infected mice lung and mouse lung epithelial cell line LA-4. (A) The protein levels of cytokines IL-1β, IL-6, RANTES and IL-10 presented in mouse lung homogenates were determined by ELISA. On day 1, 2, 4 and 6 p.i., the left lungs from infected mice (3–5 mice from each group) were homogenized in 1 ml of MEM medium. Clarified homogenates were used for cytokine detection. Non-infected mouse lung specimens were used as baseline controls. (B) mRNA level of IFN-α, IFN-β, IFN-γ and COX-2 genes in mouse lung were determined by real time RT-PCR. Mouse β-actin mRNA was used for RNA concentration normalization. Error bar indicates ±SD. * *P*<0.05. C. viral load, IL-6 and COX-2 mRNA levels in CK1- and AH1-infected mouse lung epithelial cell line LA-4 determined by real time RT-PCR. 2×10^5^ cells/per well in 12-well plate were infected with AH1 or CK1 at M.O.I of 2. At indicated times post virus infection, the cells were harvested for RNA extraction and real time RT-PCR detection of viral M gene (top panel) and IL-6 (middle panel) and COX-2 (bottom panel) mRNA. β-actin was used as RNA concentration normalization, and the data presented are the average of two experiments. * *P*<0.05.

To study the cytokine/chemokine response after CK1 infection, the pulmonary protein levels of proinflammatory cytokine interleukin-1β (IL-1β) and IL-6, anti-inflammatory cytokine interleukin-10 (IL-10), and the chemokine “regulated on activation normal T cell expressed and presumably secreted” (RANTES) were determined by enzyme immunoassay. CK1 induced high pulmonary levels of proinflammatory cytokine IL-1β, IL-6 and chemokine RANTES at all studied time points after infection (Fig. 5A). Compared to AH1, CK1 induced significantly higher IL-1β and IL-6 on day 4 p.i (*P*<0.05). The anti-inflammatory cytokine IL-10 only increased on day 6 p.i in both AH1 and CK1 infection, but at a significantly lower level in CK1-infected mice (*P*<0.05).

Since the detection of interferon production is not sensitive enough by enzyme immunoassay, we determined the mRNA levels of interferon-α (IFN-α), interferon-β (IFN-β) and interferon-γ (IFN-γ) in mouse lung homogenates using quantitative RT-PCR [21]. On day 2 p.i, more than 50-fold increase of IFN-β mRNA levels were observed in CK1-infected mice, which was significantly higher than that of AH1-infected mice (p<0.05). There was no significant difference in the IFN-α and IFN-γ mRNA levels between CK1 and AH1 (Fig. 5B).

We have previously reported that upregulation of cyclooxygenase 2 (COX-2) gene played an important role in the pathogenesis of avian H5N1 virus infection [20]. Therefore, we tested the COX-2 gene expression in mouse lungs after H7N9 infection. There was a surge of the level of COX-2 mRNA on day 1 p.i. with about 20-fold increase in CK1-infected lung tissues (Fig. 5B). However, the difference in COX-2 mRNA levels between CK1 and AH1-infected samples did not reach statistical significance.

The above findings suggest that although CK1 did not replicate as efficiently as AH1 in mouse lung, CK1 induced higher levels of proinflammatory response in infected mice than that of AH1. To test this finding, we performed an *in vitro* study using mouse lung epithelial cell line LA-4. After virus inoculation, CK1 and AH1 replicated to a comparable level in LA-4 cells (Fig. 5C, top panel), but CK1 induced significantly higher expression mRNA levels of IL-6 and COX-2 at 6 and 9 hours p.i. than AH1 (*P*<0.05).

### Combination of celecoxib and zanamivir could ameliorate lung inflammation and improve survival

**Figure 6:**
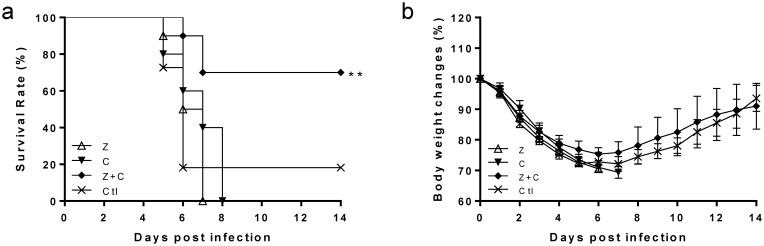
Survivals and body weight changes of mice treated with combination of zanamivir and celecoxib, zanamivir or celecoxib alone by intraperitoneal injection. The mice were infected with 10^6^ PFU of CK1 and treated with (1) combination treatment (♦): celecoxib 2 mg daily from day 2 to day 4 p.i., and zanamivir 2mg twice daily from day 2 to day 8 p.i; (2) zanamivir alone (Δ): zanamivir 2mg twice daily from day 2 to day 8 p.i; (3) celecoxib alone (▾): celecoxib 2 mg daily from day 2 to day 4 p.i., (4) control group (×): Celecoxib solven (1% DMSO/PBS) 200ul from day 2 to day 4 p.i. The survival (a) and body weight change (b) were observed for 14 days after infection. Data shown are the average of two experiments (in total, n = 10 for each treatment group, n = 17 for control group). *P<0.05 as compared to untreated control group.

**Figure 7:**
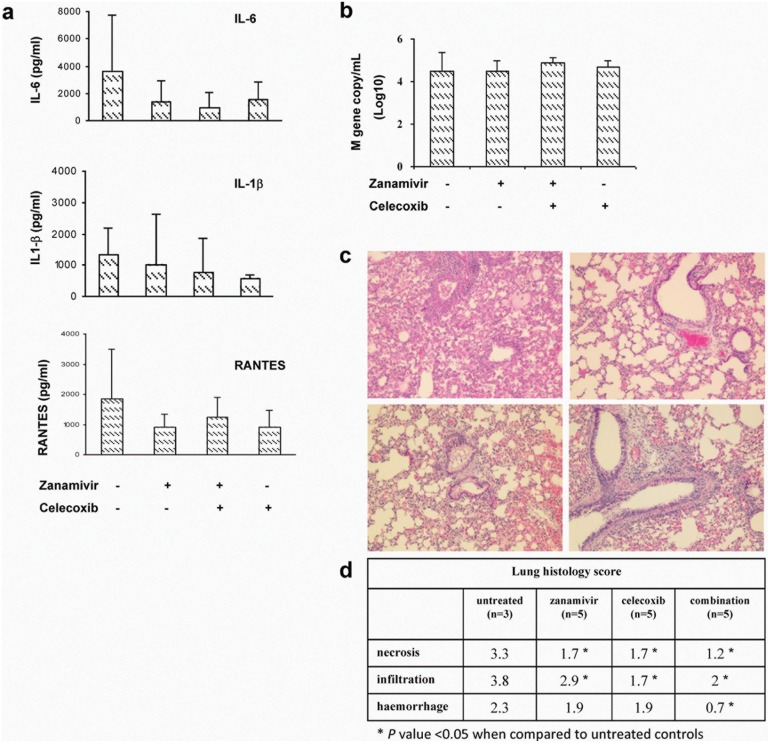
Changes of cytokines and viral loads in the lungs of CK1-infected mice after treatments. At day 4 p.i., mice from different treatment groups were sacrificed. Left-side lungs were homogenized and the clarified homogenate were used for IL-6, IL-1β and RANTES determination by EIA (a) and the viral loads were determined by real time quantitative RT-PCR (b). n = 5 for each group. Error bar indicates ±SD. (c) Representative histological images of H&E stained lung tissues of untreated mice (top left panel), treated with celecoxib-zanamivir combination (top right panel), zanamivir alone (bottom left panel), and celecoxib alone (bottom right panel). Original magnification ×100. (d) Average histology score of mouse lung tissues with different treatment.

Most H7N9 isolates are susceptible to neuraminidase inhibitors in enzymatic assay [14], [15], but the efficacy is poor in patients with delayed treatment. Mouse experiments demonstrated that neuraminidase inhibitor was only effective when administered within 24 hours after virus infection [14], [15]. We investigated whether COX-2 inhibitor celecoxib with or without neuraminidase inhibitor, zanamivir, could lower the overwhelming inflammatory responses and improve survival in mice infected with CK1 as in the case of H5N1 infection. When administered at 48 hours p.i., treatment with celecoxib-zanamivir combination had the highest survival rate, with 70% (7/10) survival and a mean survival time (MST) of 12.6 days, while the untreated control mice only had 18% (3/17) survival (*p* = 0.0059) (Fig. 6a). The survival rate of the celecoxib-zanamivir combination group was significantly higher than that of zanamivir alone (*p* = 0.0013) or celecoxib alone (*p* = 0.0037). All the mice in the zanamivir alone group and celecoxib alone group succumbed to the infection. At day 4 p.i., all three treatment groups (zanamivir and celecoxib combination, zanamivir alone or celecoxib alone group) showed a trend of reduction in the pulmonary levels of proinflammatory cytokine IL-6, IL-1β and RANTES although this reduction was not statistically significant (Fig. 7a). There was no difference in the pulmonary viral load among all groups at day 4 p.i. (Fig. 7b). Compared with the diffuse alveolar infiltration and exudation in untreated mice (Fig. 7c), mice in the celecoxib-zanamivir combination group showed mainly mild bronchiolitis with peribronchiolar lymphocytic infiltration, adjacent mild alveolitis and vascular congestion. Pathological changes were also ameliorated with zanamivir and/or celecoxib treatment (Fig. 7d).

## Discussion

The 2013 H7N9 viruses isolated from poultry and human in China are closely related phylogenetically [1], [22], but H7N9 virus isolated from poultries often lacks important genetic signature for mammalian adaptation. For example, CK1 did not possess either the PB2 627K or 701N mutation, which is found in most H7N9 isolates from human [1], [3]. In this study, we assessed the virulence of CK1 using a well-established influenza mouse model [20], [23]–[25]. We have shown that CK1 could cause lethal infection in mice, and the MLD_50_ was <2 log higher than that of the human H7N9 virus AH1. Mice infected with 10^6^ PFU of CK1 exhibited similar mortality rate, body weight loss, and pulmonary damage as those infected with 10^5^ PFU of AH1. Our data suggests that CK1 may also be virulent in humans without further adaptations. CK1 may be more virulent in mice than other poultry H7N9 virus isolates tested in other studies. Zhang *et al* compared the virulence of three poultry isolates of 2013 H7N9 virus (A/chicken/Shanghai/S1053/2013, A/pigeon/Shanghai/S1069/2013 and A/pigeon/Shanghai/S1421/2013) with AH1 [18]. Mice inoculated with ≥10^4^ 50% egg infectious dose of AH1 had significant weight loss; while mice inoculated with the chicken or pigeon H7N9 isolates did not have any weight loss. Similarly, Belser *et al* inoculated mice with A/shoveler/Egypt/00215-NAMRU3/07 at a dose >100 times higher than the MLD_50_ for AH1, but no mice died [17]. Watanabe *et al* found that the MLD_50_ was about 3 logs higher for A/duck/Gunma/466/2011 (H7N9) than that of AH1 [14]. When compared to three poultry isolates with low virulence in mice, there are several unique substitutions found in CK1 in the haemagglutinin, neuraminidase and NS1 protein (table S1). For instance, CK1 HA gene contains avian type 217Q (226 in H3 numbering) rather than the 217L that is found among the majority of H7N9 human and avian isolates. This may favour the infection in mouse respiratory tract which predominantly expresses α-2,3 linked sialic acids receptors. Future studies comparing CK1 with the other poultry H7N9 viruses with low virulence in mice may help us to understand specific mutations that contribute to high virulence in mammals.

Immunohistochemical staining with anti-NP antibody showed that, similar to AH1, CK1 infected the middle and lower respiratory tract. A similar phenomenon was seen in H5N1 viruses originating from Hong Kong, in which both avian and human viruses led to similar extent of infection in the respiratory tract of mice [26]. On the other hand, the pulmonary viral loads were higher in the AH1 group than the CK1 group even inoculation dose of CK1 was 10-time higher than AH1, suggesting that AH1 replicates better in the mammalian respiratory tract than CK1. One possibility is that CK1 can induce a higher level of IFN-β than AH1, which may suppress the viral replication better. Another possibility is that CK1 lacks the PB2 E627K substitution which is associated with more efficient replication at a lower body temperature, a characteristic of mammalian adaptation when compared with the situation in avian species [1], [27], [28]. This finding is also similar to that of H5N1 virus, in which strains with PB2 627K can replicate to a higher titre in mice lungs than strains with PB2 627E [29], [30].

Cytokine dysregulation is a feature of severe influenza in humans and animal models. Patients with severe H5N1 and 2009 pandemic H1N1 had very high levels of IL-6 [31], [32]. Our previous study comparing the wild type 2009 pandemic H1N1 and its more virulent mutant with D225G substitution in the haemagglutinin showed that the levels of IL-6 and IL-1β were higher in mice infected with mutant virus than those with wild type virus [24]. CK1 had a significantly lower level of pulmonary viral load when compared to AH1-infected mice, but CK1 induced a more pronounced proinflammatory cytokine/chemokine response. One exception is that CK1 had a lower IL-10 level than that of AH1 on day 6 p.i. In another study comparing different 2009 pandemic H1N1 isolates in mice, more virulent viral isolates induced higher levels of IL-6, but lower levels of IL-10 [33]. As the viral load was lower in the CK1 group, the difference in cytokine/chemokine cannot be explained by the difference in the initial viral inoculation dose. In human, more severe cytokine dysregulation has been demonstrated in fatal than non-fatal cases of influenza virus infection [1], [31]. NS1 has been shown to be important in the induction of host inflammatory response [34]. NS1 may affect the innate immune response through the interaction between RIG-I [35] and host proteins containing the PDZ domain [36]. There is one amino acid difference in the NS1 proteins of AH1 and CK1 at amino acid position 3 (serine in AH1 and phenylalanine in CK1), but the significance of this difference remains to be determined.

The type of histopathological changes induced by CK1 was similar to that of AH1. Extrapulmonary involvement in infected mice was limited to histopathological changes without virological evidence of direct infection which is consistent with AH1 infection in ferrets [37]. The degenerative changes in the heart, liver and kidney during the later stage of infection may be related to the hypoxic and immune-related damage triggered by the cytokine dysregulation as seen in severe influenza [1], [24], [31], [32]. In ferrets, AH1 could cause hepatic lipidosis [37]. The lack of extrapulmonary viral dissemination is compatible with our recent clinical study showing lack of viremia and absence of viruses in tissues from patients [5]. This is in contrast to H5N1 infection, in which extrapulmonary spread is commonly seen in mouse models [38]. One major characteristic of H5N1 virus is the presence of multibasic amino acid at the cleavage site of HA, which renders it susceptible to cleavage by a wide range of tissue proteases [7], whereas H7N9 virus with only one arginine at this cleavage site may have limited its tropism to the respiratory tissues [1], [2]. The lack of extrapulmonary spread in our mouse model is similar to another mouse model using SH2 [19], but different from the ferret model, in which SH2 was detected outside the respiratory tract, including the central nervous system[39]. However, the difference in virus strain and animal host makes a direct comparison difficult. In another study, AH1 caused disseminated infection in mouse, but the infectious dose (10^6-8^ TCID_50_) was higher than that in our study [16].

Celecoxib, a COX-2 inhibitor, reduced pulmonary inflammation and improved survival in this study when combined with zanamivir despite their delayed administration at 48 hr p.i. which is similar to a previous study of H5N1 infection [20]. The beneficial effect of the addition of celecoxib may also be related to the suppression of viral replication [40]. However, the cytokine/chemokine profile and viral loads were similar to zanamivir alone, celecoxib alone, or even the untreated control group at day 4 p.i. Our results are similar to that of another study using oseltamivir in the treatment of H7N9 infection in mice [15]. The lack of reduction in the pulmonary viral loads suggests that our treatment regimens had little antiviral effect in the lungs during the early period of infection. This indicated other mechanisms may be involved in the improvement of the survival of CK1-infected mice. Currently, there are no human studies on the use of celecoxib despite its frequent clinical use for many years as an anti-inflammatory medication. As the mortality remains high despite the use of neuraminidase inhibitor in H7N9 infections and that some strains of H7N9 viruses are resistant to neuraminidase inhibitors [1], [2], [14], celecoxib-zanamivir combination should be further investigated in randomized clinical trials for treating severe influenza.

In this study, we have used zanamivir instead of oseltamivir in the treatment experiments for several reasons. Firstly, zanamivir has low IC_50_ against H7N9 virus, even for those with R294K mutations which confer resistance to oseltamivir [9], [14]. Secondly zanamivir can be given intravenously in humans, while oseltamivir can only be given via the oral route or through nasogastric tubes which may not be feasible in some patients with severe H7N9 infection. Furthermore, intravenous route is not affected by absorption in the gastrointestinal tract. Intravenous zanamivir also results in a very high systemic drug concentration. After one single dose of 600 mg intravenous zanamivir, the C_max_ is above 30 µg/ml, which is much higher than the IC_50_ of H7N9 virus, even for strains with R294K mutations [41]. Intravenous zanamivir has been used in H7N9 patients with favourable outcome [11]. We used the intraperitioneal route in mice to mimic the intravenous route in humans.

There are several limitations to our study. Firstly only one chicken isolate of H7N9 was studied. Further studies should be performed for more poultry viral isolates with and without epidemiological link to human cases. Secondly, although the mouse model is a well-established mammalian model for influenza, important differences exist between mice and humans which may affect clinical relevance.

### Conclusion

We have demonstrated that chicken H7N9 virus can cause lethal disease in mice, which was associated with severe pulmonary and extrapulmonary pathologies, and cytokine dysregulation. Although the chicken H7N9 isolate had a lower viral replication in the lung, it has triggered a more intense proinflammatory cytokine/chemokine response. Celecoxib and zanamivir appear to ameliorate pulmonary inflammation and improve survival.

## Materials and Methods

### Viral isolates and animals

The H7N9 virus CK1, isolated from a chicken, and AH1, isolated from a fatal human case, were used in this study [1], [2]. The viruses were propagated in 10-day-old specific-pathogen-free (SPF) chicken embryos at 37°C for 48 hours as described previously without serial passage. Allantoic fluid was titrated in MDCK cells for the determination of TCID_50_ and PFU as described previously [42]. Aliquots of virus stock were stored at −80°C until use. HA, NA, PB2 and NS genes of the CK1 stock were sequenced and found no additional mutations other than the sequence data deposited in the GeneBank. Female BALB/c mice at 6–8 weeks old were obtained from the Laboratory Animal Unit, the University of Hong Kong. The animals were housed in SPF-free facilities with 12-hour light-dark cycles and standard pellet feed and water *ad libitum*. Virus challenge experiments were carried out in biosafety level 3 animal facilities. All animal-related experiments were performed according to the standard operating procedures as previously described [20] and were approved by the University of Hong Kong committee on the use of live Animals in teaching and research.

### Virus inoculation and drug treatment

For the determination of MLD_50_, groups of six BALB/c mice were inoculated with 50 µL 10-fold dilutions of CK1 or AH1 via intranasal route under ketamine (100 mg/kg given intraperitoneally) and xylazine (10 mg/kg given intraperitoneally) anaesthesia. Mortality was observed for 14 days. MLD_50_ titres were calculated by the method of Reed and Muench. For pathogenesis study, groups of BALB/c mice were inoculated via intranasal route with 10^6^ PFU of CK1 or 10^5^ PFU of AH1 under ketamine (100 mg/kg) and xylazine (10 mg/kg) anaesthesia. The body weights, symptoms, and survivals of the mice were monitored daily for 14 days after virus inoculation. Disease severity was scored (Table S2). As a humane endpoint, the animals were euthanized by intraperitoneal injection of pentobarbital sodium (100mg/kg) when the disease score was 4, or when the disease score was 3 with a weight loss exceeding 30%. For sample collection on 1, 2, 4 and 6 days p.i., three to five mice from each group were sacrificed to collect their blood, brain, heart, liver, kidneys and lungs. The collected organs were separated into two sets, one set being frozen at −80°C for RNA and protein extraction, and the other set being fixed in 10% neutral formalin for histopathological study. Drug treatment was modified from our previous study for H5N1 [20]. Celecoxib (Sigma-Aldrich, St. Louis, MO, USA) was dissolved in 10% dimethyl sulfoxide at 100 mg/ml; and zanamivir (GlaxoSmithKline Australia Pty LTD, Boronia, Australia) was dissolved in phosphate buffered saline (PBS) at 10 mg/ml. Mice were inoculated with 10^6^ PFU of CK1. Ten mice in each treatment group and 17 in control group were observed for 14 days to monitor survival. An additional five mice in each treatment group and 3 mice in control group were sacrificed on day 4 p.i. for pathological study. The celecoxib-zanamivir combination group was treated with intraperitoneal injection of celecoxib at 2 mg/day from day 2 to day 4 p.i. and intraperitoneal injection of zanamivir 2 mg twice daily from day 2 to day 8 p.i. The zanamivir alone group was treated with zanamivir twice daily from day 2 to day 8 p.i. Celecoxib alone group was treated with celecoxib at 2 mg/day from day 2 to day 4 p.i. Untreated control group received same volume of solvents for celecoxib.

### Determination of viral load in homogenized specimens

The left lungs, left hemispheres of the brain, left kidneys, and 0.2 g of liver were homogenized separately in 1 ml of cold minimum essential medium (MEM) supplemented with 1% penicillin and streptomycin. Clarified samples of homogenate supernatant were stored in aliquots at −80°C until use. For quantitative real-time RT-PCR detection of viral gene expression, total RNA were extracted from 350 µl of clarified tissue homogenates using Qiagen RNeasy Mini kit (Qiagen, Germantown, MD, USA) as we described previously [20], [23]. Reverse transcription was performed using Superscript RT II enzyme (Life Technology, CA, USA) using influenza specific UNI12 primer (5′-AGCAAAAGCAGG-3′). The cDNA was amplified by real-time PCR performed on LightCycler 480 system (Roche Applied Sciences) using SYBR Green I Master (Roche). Influenza A M gene was used as the target gene with forward primer 5′-CTTCTAACCGAGGTCGAAACG-3′ and reverse primer 5′-GGCATTTTGGACAAAKCGTCTA-3′. The pcDNA3.1 plasmid containing the cloned M gene fragment was applied as standard. The detection limit of this assay was 100 copies of the viral M gene per ml of tissue homogenates. Viral titres were also determined by TCID_50_ assay as we described previously [42].

### Determination of interferons and cytokines in homogenized lung specimens

The pulmonary expression levels of IFN-α, IFN-β, IFN-γ, and COX-2 mRNA were determined by real-time RT-PCR using oligodT transcribed-cDNA from lung tissue homogenates. The expression of β-actin was quantified by real-time RT-PCR and used for RNA normalization, and a ΔΔCt method was used to estimate the differential gene expression between samples. Primers for real-time PCR: IFN-α forward primer: 5′-ARSYTGTSTGATGCARCAGGT-3′, IFN-α reverse primer: 5′-GGWACACAGTGATCCTGTGG; IFN-β-forward primer: 5′-AGCTCCAAGAAAGGACGAACAT-3′, IFN-β-reverse primer, 5′-GCCCTGTAGGTGAGGTTGATCT-3′; IFN-γ-forward primer: 5′-ARSYTGTSTGATGCARCAGGT-3′, IFN-γ-reverse primer: 5′-GGWACACAGTGATCCTGTGG-3′; Mouse β-actin-forward primer: 5′-TCACCCACACTGTGCCCATCTACGA-3′, Mouse β-actin-reverse primer: 5′-GGATGCCACAGGATTCCATACCCA-3′; COX-2 forward primer: 5′-TCTGGAACATTGTGAACAACATC-3′, reverse primer: 5′-AAGCTCCTTATTTCCCTTCACAC-3′.

Protein levels of IL-1β, IL-6, IL-10, and RANTES in clarified lung homogenates were determined by enzyme immunoassay (R&D system, Inc., Minneapolis, MN, USA) as described previously [23]. The detection limits of these assays were 7.8 pg/ml for IL-1β, IL-6 and RANTES, and 16 pg/ml for IL-10.

### Histopathological and immunohistochemical staining of influenza nucleoprotein in lung tissue

To examine pathological changes of infected mouse lung at different time after infection, right side of the lung were fixed in 10% formalin. All 4 lung lobes were embedded in paraffin and sectioned at 5µm for haematoxylin and eosin (H&E) staining. All lung fields of the 4 lobes were examined at 20x magnification for each sample. The severity of histological changes was graded according to a semiquantitative scoring system (Table 2) [17], [43], [44].

**Table 2:** Mouse lung tissue histological score.

Histological changes	Airway and alveolarcells necrosis (necrosis)	Cell infiltration and alveolarhyaline membraneformation (infiltration)	Alveolar hemorrhage(hemorrhage)
**Score 0**	Normal lung	Normal lung	Normal lung
**Score 1**	Airway epithelial cellnecrosis limited in onelung lobe	Infiltration cells only onvessel wall or peribronchiolarin one lobe	Hemorrhage restrictedin one small area (one 20x field)
**Score 2**	Airway epithelial cellnecrosis in more than one lunglobes, with cell debris congestedin airway lumens	Few cells (1–5 cells) in airspace but in focal areaof one lobe	Hemorrhage in one larger area(more than one 20x field)in one lobe
**Score 3**	Airway epithelial cell necrosisin more than one lung lobes;small area of alveolar wallcollapse	More cells in air space in morethan one lobe; and alveolar spacehyaline membrane formation infocal area	Hemorrhage in more than onelung lobes, with focal area ofalveolar space congested with RBC
**Score 4**	Airway epithelial cell necrosisin more than one lung lobesalveolar wall collapse in morethan one lobes	Severe infiltration with airspace congested; large areaof hyaline formation in morethat one lobes	All 4 lobes showed focalor diffuse hemorrhage

For influenza NP staining, de-paraffinized and rehydrated tissue sections were treated with Antigen Unmasking Solution according the manufacturer’s instructions (Vector Laboratories Inc. Burlingame, CA, USA) to unmask the antigens. After blocking with 1% bovine serum albumin, the sections were incubated with mouse anti-influenza NP-antibody (HB65, ATCC) at 4°C overnight, followed by biotin-conjugated goat anti-mouse IgG (Calbiochem, Darmstadt, Germany) for 30 min at room temperature. Streptavidin/peroxidase complex reagent (Vector Laboratories, Burlingame, CA) was then added and incubated at room temperature for 30 min. Colour development was performed with 3, 3′-diaminobenzidine (DAB, Vector Laboratories, Burlingame, CA, USA) and images were captured with Nikon80i imaging system with the help of Spot-advance computer software.

### *In vitro* infection of mouse lung epithelial cells and detection of viral load, IL-6 and COX-2 mRNA level by quantitative real time RT-PCR

Mouse lung epithelial cell line LA-4 (ATCC # CCL-196) was seeded in 12-well plates at 2×10^5^ cells per well and incubated overnight at 37°C with 5% CO_2_. The cells were incubated with CK1 or AH1 at a multiplicity of infection of 2 for 1 h at 37°C. The cells were washed twice with PBS after removing the virus,and further incubated for 1, 3, 6, and 9 hours. At each time point after infection, the cells were harvested. Total RNA extraction, cDNA transcription and real-time RT-PCR determination of viral M gene copies, IL-6 and COX-2 mRNA level were performed as described above.

### Statistical analysis

Mouse survival rates were analyzed by the Kaplan-Meier method and Log-rank test using SPSS 17.0 for Windows (SPSS Inc., Chicago, IL). Pulmonary viral loads, cytokine, chemokine profiles, and histology scores were analyzed by Student’s t-test. A *P* value of <0.05 was considered statistically significant.

## Supporting Information

Table S1 **Unique substitutions in sequence of CK1 virus when compared to other H7N9 poultry isolates with low pathogenicity in mice.**
(DOC)

Table S2 **H7N9 infected mouse disease severity scoring system.**
(DOC)
