# Supplementary material for: Avian Influenza A H7N9 Virus Induces Severe Pneumonia in Mice without Prior Adaptation and Responds to a Combination of Zanamivir and COX-2 Inhibitor
Source: PLoS One. 2014 Sep 18;9(9):e107966. doi: 10.1371/journal.pone.0107966 (PMC4169509; doi:10.1371/journal.pone.0107966)
Supplement: Table S1 — Unique substitutions in sequence of CK1 virus when compared to other H7N9 poultry isolates with low pathogenicity in mice. (DOC) [file pone.0107966.s001.doc]

**Table S1. Unique substitutions in CK1 when compared to poultry isolates with low pathogenicity in mice.**

|  | CK1 | S1053 | S1069 | S1421 |
| --- | --- | --- | --- | --- |
| HA (H7 numbering) |  |  |  |  |
| 24 | G | V | V | V |
| 47 | K | P | P | P |
| 54 | G | C | C | C |
| 56 | P | L | L | L |
| 57 | R | L | L | L |
| 78 | M | I | I | I |
| 217 | Q | L | L | L |
| NA (N9 numbering) |  |  |  |  |
| 423 | V | L | L | L |
| 436 | A | T | T | T |
| 437 | G | S | S | S |
| NS1 or NS2 |  |  |  |  |
| 3 | F | S | S | S |
|  |  |  |  |  |

S1053, A/chicken/Shanghai/S1053/2013;

S1069, A/pigeon/Shanghai/S1069/2013;

S1421, A/pigeon/Shanghai/S1421/2013
