# Supplementary material for: Avian Influenza A H7N9 Virus Induces Severe Pneumonia in Mice without Prior Adaptation and Responds to a Combination of Zanamivir and COX-2 Inhibitor
Source: PLoS One. 2014 Sep 18;9(9):e107966. doi: 10.1371/journal.pone.0107966 (PMC4169509; doi:10.1371/journal.pone.0107966)
Supplement: Table S2 — H7N9 infected mouse disease severity scoring system. (DOC) [file pone.0107966.s002.doc]

**Table S2.** Disease severity scoring system.

| Disease severity score | Symptoms |
| --- | --- |
| 0 (Apparently healthy) | Nil |
| 1 (Mild disease  symptom) | Ruffled fur, but still active |
| 2 (Moderate disease symptom) | Ruffled fur + reduced activity + no weight gain |
| 3 (Severe disease symptom) | Ruffled fur + hunched posture + labored breathing + weight loss |
| 4 (Moribund) | Very inactive, showing difficulty moving around and accessing to food and water + weight loss |
